# Distinct bacterial community structures and arsenic biotransformation gene profiles in dust

**DOI:** 10.3389/fmicb.2025.1607082

**Published:** 2025-07-30

**Authors:** Yi Yin, Yu-Ting Lin, Gong-Ren Hu, Rui-Lian Yu, Xiao-Hui Sun, Yu Yan

**Affiliations:** ^1^Department of Environmental Science and Engineering, College of Chemical Engineering, Huaqiao University, Xiamen, China; ^2^Department of Bioengineering and Biotechnology, College of Chemical Engineering, Huaqiao University, Xiamen, China

**Keywords:** arsenic, arsenic biotransformation genes, bacterial communities, dust, metagenomes

## Abstract

**Introduction:**

Microorganisms, which are ubiquitous in the environment, have evolved a diverse array of arsenic biotransformation genes (ABGs). Dust harbors a wide range of microorganisms. However, the distinct characteristics of bacterial community structures and ABG profiles in dust, compared with those in other environments such as soil and water, remain poorly understood.

**Methods:**

In this study, dust samples were simultaneously collected alongside surrounding soil and seawater samples in Xiamen, a coastal city of China, to investigate the distinct profiles and potential sources of bacterial communities and ABGs in dust using 16S rRNA gene amplicon sequencing and metagenomic sequencing.

**Results and discussion:**

Abundant and diverse bacterial communities and ABGs were detected in dust, revealing significant differences in community structures and ABG profiles compared with those in soil and seawater. Soil was identified as the primary source for both bacterial communities and ABGs in dust through fast expectation–maximization microbial source tracking (FEAST). Acetobacteraceae, which showed significantly greater relative abundance (*p* < 0.001) in dust than in soil and seawater, was also identified as a keystone taxon in the dust bacterial co-occurrence network. Furthermore, metagenome-assembled genomes (MAGs) affiliated with Acetobacteraceae were effectively recovered from dust via metagenomic binning, and these MAGs harbored an array of ABGs, indicating that Acetobacteraceae could be important hosts for ABGs in dust. Overall, our findings offer new insights into bacterial communities and ABGs in dust, thereby improving our understanding of arsenic biogeochemical cycling.

## 1 Introduction

Arsenic, a widely distributed toxic metalloid in various environments, originates from both natural processes and human activities (Bowell et al., 2014; Huang et al., 2024). Arsenic contamination poses a considerable risk to both ecosystems and human health, thereby posing a global environmental concern (Chen and Costa, 2021; Tuli et al., 2010). The main concern regarding arsenic contamination has traditionally been associated with its presence in groundwater, surface water, soil, sediment, and biota (Bhattacharya et al., 2002; Raju, 2022). However, arsenic in the air, which exists primarily (> 90%) in particulate form, could also potentially impact the health of the population (Sánchez-Rodas et al., 2015). Dust, a prominent component of atmospheric particulate matter, can facilitate the transport of arsenic (Yin et al., 2013). The total concentration of arsenic in dust and the associated risks have been extensively studied (Balakrishna et al., 2011; Ma et al., 2021). For example, the arsenic concentrations in dust samples collected from kindergartens in Xiamen, China, were found to be approximately 2.2 times greater than the background value of the soil (Ma et al., 2021). Furthermore, the toxicity of arsenic is closely linked to its chemical speciation (Jain and Ali, 2000). The speciation of arsenic in atmospheric particulates, including dust, has also been analyzed recently (Sánchez-Rodas et al., 2015; Xie et al., 2021; Yan et al., 2024; Yin et al., 2017). Generally, the predominant species of arsenic found in dust is inorganic arsenate [As(V)]. In addition, other species, such as inorganic arsenite [As(III)], trimethylarsine oxide [TMAs(V)O], and dimethylarsenate [DMAs(V)], have also been identified in dust.

Microorganisms are ubiquitous in the environment. In addition to soil and seawater, air also serves as a crucial reservoir of microorganisms. Numerous studies have demonstrated that microorganisms are key components of dust (Ding et al., 2020; Leppänen et al., 2018). Moreover, microorganisms can drive the biogeochemical cycling of arsenic, and arsenic biotransformation genes (ABGs) are widespread in microorganisms owing to their longstanding coexistence with arsenic (Ben Fekih et al., 2018; Mukhopadhyay et al., 2002; Oremland and Stolz, 2003). For example, the redox transformation of arsenic can be achieved through As(V) reduction mediated by genes encoding cytoplasmic As(V) reductases (ArsC) or respiratory As(V) reductases (ArrAB) and the oxidation of As(III) mediated by genes encoding As(III) oxidases (AioAB/ArxAB) (Andres and Bertin, 2016; Oremland and Stolz, 2005). Arsenic methylation and demethylation can be facilitated by genes encoding As(III) *S*-adenosylmethionine methyltransferases (ArsM) and C-As lyases (ArsI), respectively (Qin et al., 2006; Yoshinaga and Rosen, 2014). The distribution and abundance of ABGs have been extensively studied in water, soil, and sediment through the utilization of quantitative PCR (qPCR) (Guo et al., 2019; Qiao et al., 2023; Zecchin et al., 2021; Zhang et al., 2015). In particular, our recent study revealed the presence of abundant ABGs (e.g., the *arsM*, *arsC*, and *aioA* genes) in dust for the first time by using a high-throughput qPCR chip designed for detecting ABGs (Yan et al., 2024; Zhao et al., 2019). Although qPCR method is extensively used for arsenic-related gene detection, this method could only target a limited number of specific ABGs and was prone to non-specific amplification introduced by the primers due to primer design constraints (Song et al., 2022). While qPCR can determine the abundance of ABGs, it cannot provide information on their taxonomic compositions. Metagenomic sequencing technology enables the simultaneous probing of both the abundance and taxonomic profiling of numerous ABGs, thereby characterizing microbial-driven arsenic biotransformation at gene- and species-level resolutions (Li L. et al., 2023). Therefore, metagenomic sequencing can be further employed to elucidate ABGs in the environment more comprehensively. Furthermore, airborne and dust-associated microbial communities can originate from other environments, such as soil and water (Archer et al., 2023; Barberan et al., 2015; Bowers et al., 2011). However, the differences in bacterial community structures and ABG profiles in dust compared with those in other environments, including soil and water, as well as the extent of contribution from these environments to the sources of bacteria and ABGs in dust, still require further investigation.

In this study, we simultaneously collected dust samples and surrounding soil and seawater samples in Xiamen, China, to systematically characterize the distinct profiles and potential sources of bacterial communities and ABGs in dust. This study aimed to (i) characterize the variations in bacterial community structures and ABG profiles across dust, soil, and seawater; (ii) track the sources of bacterial communities and ABGs in dust; and (iii) identify key bacterial taxa and ABGs in dust.

## 2 Materials and methods

### 2.1 Sample collection and pretreatment

**Figure 1:**
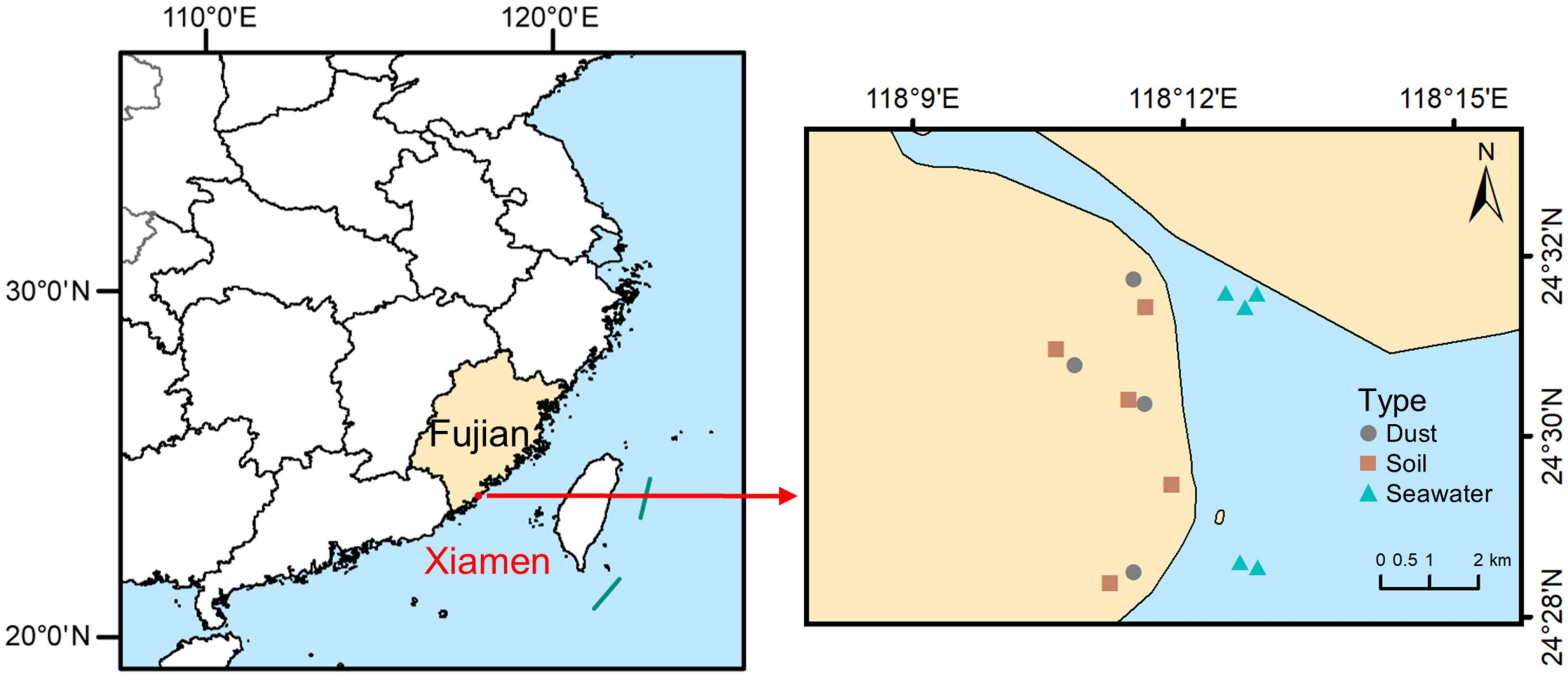
Study area and sampling sites.

Xiamen (24°23′N to 24°54′N and 117°52′E to 118°26′E), situated on the southeastern coast of China, is a typical coastal city where the interaction between land and sea creates an environment in which dust, soil, and seawater are closely interconnected. Samples of dust, soil, and seawater were collected from coastal parks situated in Xiamen in September 2023 during a dry period with no rainfall for at least seven consecutive days. The locations of the sampling sites are illustrated in Figure 1. Dust samples were collected by manually sweeping outdoor surfaces with sterilized brushes. Soil samples were collected from a depth of 0–10 cm via a sterile sampling shovel. Seawater samples were collected at depths of 20–30 cm below the sea surface using polyethylene bottles. After thorough mixing, each sample of dust and soil was separated into two parts: one part was air-dried, sieved (<150 μm), and stored at room temperature for determining the total concentrations and speciation of arsenic, while the other portion was frozen at −20°C to facilitate DNA extraction. Each seawater sample was also separated into two parts: one part was acidified with HNO_3_ for determining the total concentration of arsenic, while the other part was filtered with 0.22 μm mixed cellulose ester (MCE) membranes to collect the microorganisms, after which the membranes were placed in sterile freezing tubes and frozen at −20°C to facilitate DNA extraction.

### 2.2 Determination of total arsenic concentrations and arsenic species

The dust and soil samples were digested with a mixture of HNO_3_ and HCl (3:1, v/v) at 180°C for 24 h (Ma et al., 2021). The digested samples were diluted with 2% (v/v) HNO_3_, and the acidified seawater samples were then filtered through 0.45 μm filters. The total concentration of arsenic was determined via inductively coupled plasma-mass spectrometry (ICP-MS; 7800, Agilent Technologies, United States) at the Instrumental Analysis Center of Huaqiao University. For quality control, a certified reference material (GSS-5) was used, with recovery rates of arsenic ranging from 97.6% to 111.5%.

The dust and soil samples were extracted with 0.3 M H_3_PO_4_ at a solid–liquid ratio of 1:200 (w/v) for 30 min at 150 rpm and 25°C, followed by an additional sonication step for 30 min (Orero Iserte et al., 2004). Both extracts were filtered through 0.45 μm filters. Arsenic species were determined via high-performance liquid chromatography (HPLC, 1260 II, Agilent Technologies, United States) couple with ICP-MS (7800, Agilent Technologies, USA) at the Instrumental Analysis Center of Huaqiao University. A PRP-X100 anion-exchange column (250 mm × 4.1 mm, 10 μm, Hamilton, United States) was used for analysis. The mobile phase consisted of a mixture of 10 mM NH_4_NO_3_ and 10 mM NH_4_H_2_PO_4_ adjusted to pH 6.2 with a flow rate of 1 mL·min^−1^ (Yin et al., 2011; Yan et al., 2024). The arsenic species in the seawater samples were not determined owing to the low total arsenic concentrations in these samples. For quality control, spike recovery experiments were performed with known amounts of arsenic species spiked to the raw samples (Kara et al., 2021; Pizarro et al., 2003), yielding recovery rates between 86.9% and 97.3% for each individual arsenic species.

### 2.3 DNA extraction and high-throughput 16S rRNA gene sequencing

Total microbial genomic DNA was extracted from the dust and soil samples as well as the filters that captured microorganisms from the seawater samples via a FastDNA Spin Kit for Soil (MP Biomedicals, United States). The extracted DNA was quantified via a Qubit 3.0 fluorometer (Invitrogen, Belgium). The bacterial 16S rRNA gene (V4-V5 region) was amplified via the 515F/907R primers (Ma et al., 2023). Amplicon sequencing was performed on the Illumina NovaSeq-PE250 platform at Shanghai Personal Biotechnology Co., Ltd., China. High-quality sequences were processed via USEARCH (v.11.0.667) (Edgar, 2010) and subsequently clustered into amplicon sequence variants (ASVs) via UNOISE3 (Edgar, 2016). ASV classification was performed with the Silva database (v. 138) (Quast et al., 2013).

### 2.4 Metagenomic sequencing

Dust, soil, and seawater DNA were used for library preparation and shotgun metagenomic sequencing. The Illumina NovaSeq platform at Shanghai Personal Biotechnology Co., Ltd., China, was utilized for paired-end sequencing. Approximately 10 Gb of raw sequencing data was generated for each sample and subsequently filtered, subjected to quality control, and trimmed via Fastp (v0.21.0) (Chen et al., 2018b). The clean reads were individually assembled into contigs via Megahit (v.1.2.9) with default parameters, and the quality of the assembly was assessed via Quast (v5.2.0) (Gurevich et al., 2013; Li et al., 2015). Contigs (≥ 200 bp) were translated into protein-coding open reading frames (ORFs) via Prodigal (v.2.6.3) (Hyatt et al., 2010). A non-redundant gene catalog was generated by clustering ORFs at a sequence identity of 90% and a query coverage of 95% via CD-HIT (v4.8.1) (Li and Godzik, 2006). The ABGs were annotated by searching against the prebuilt arsenic biotransformation gene database (AsgeneDB) (Song et al., 2022) using Diamond (Buchfink et al., 2015) with a query coverage ≥ 80% and an identity threshold ≥ 50%. The abundance of ABGs was quantified via Salmon (v0.13.1) (Patro et al., 2017) and normalized to genes per million (GPM) based on the gene length and sequencing depth (Shaffer et al., 2020; Zhang et al., 2023). The taxonomic compositions of ABGs were generated using the “Asgene” R package.^1^

Furthermore, the clean reads obtained from dust, soil, and seawater samples were co-assembled via Megahit (v.1.2.9) (Li et al., 2015), and the co-assembled contigs were binned into metagenome-assembled genomes (MAGs) via the MetaWRAP pipeline (v1.3.2) (Uritskiy et al., 2018). The resulting MAGs were subjected to further refinement through MetaWRAP and were subsequently dereplicated via dRep (v3.5.0) (Olm et al., 2017). The assessment of MAG quality was conducted using CheckM (v.1.0.12), and only MAGs with high completeness (> 80%) and low contamination (< 10%) were retained for further analysis (Parks et al., 2015). The quantification of the relative abundance and the taxonomic annotation for each MAG were carried out using CoverM (v0.4.0) and the Genome Taxonomy Database Toolkit (GTDB-Tk v2.1.0), respectively (Chaumeil et al., 2019). Both a BLASTP-based search and a hidden Markov model (HMM)-based search were used for ABG annotation in the MAGs (Lavy et al., 2019; Song et al., 2022).

### 2.5 Statistical analyses

Alpha and beta diversity analyses were performed via the R ‘vegan’ package (v2.5–7) (Oksanen et al., 2011). The sources of the bacterial communities and ABGs in the dust samples were tracked using fast expectation–maximization microbial source tracking (FEAST), a highly efficient community-based microbial source tracking method that employs an expectation–maximization algorithm and demonstrates significant advantages in both computational efficiency and estimation accuracy (Shenhav et al., 2019). The FEAST analysis was performed via the R ‘FEAST’ package (v0.1.0). Key bacterial taxa and ABGs indicative of the different environments were identified by random forest classification models via the R ‘randomForest’ package (v4.6–14) (Liaw and Wiener, 2002). Co-occurrence networks depicting interactions between bacterial communities (ASVs) and ABGs were constructed via Spearman correlation analysis (|r| > 0.50 and *p* < 0.05) and visualized via Gephi (v0.10.1) (Bastian et al., 2009). Most of the figures were generated via Origin 2024.

## 3 Results

### 3.1 Total concentrations and speciation of arsenic

The concentrations of total arsenic in the dust, soil, and seawater samples ranged from 8.49 to 57.18 mg·kg^−1^, 3.18 to 10.41 mg·kg^−1^, and 1.50 to 2.49 μg·L^−1^, respectively (Supplementary Table 1). Additionally, the coefficient of variation (CV) for arsenic in the dust samples was markedly greater at 97.48%, whereas it was 37.99% in the soil samples and 17.43% in the seawater samples. Inorganic arsenic species [As(V) and As(III)] constituted more than 95% of the total arsenic species detected in dust and soil samples (Supplementary Table 2). Furthermore, in dust samples, TMAs(V)O was the main methylated arsenic species, whereas in soil samples, MAs(V) and DMAs(V) were the predominant species identified.

### 3.2 Variations in bacterial community structures and ABG profiles

**Figure 2:**
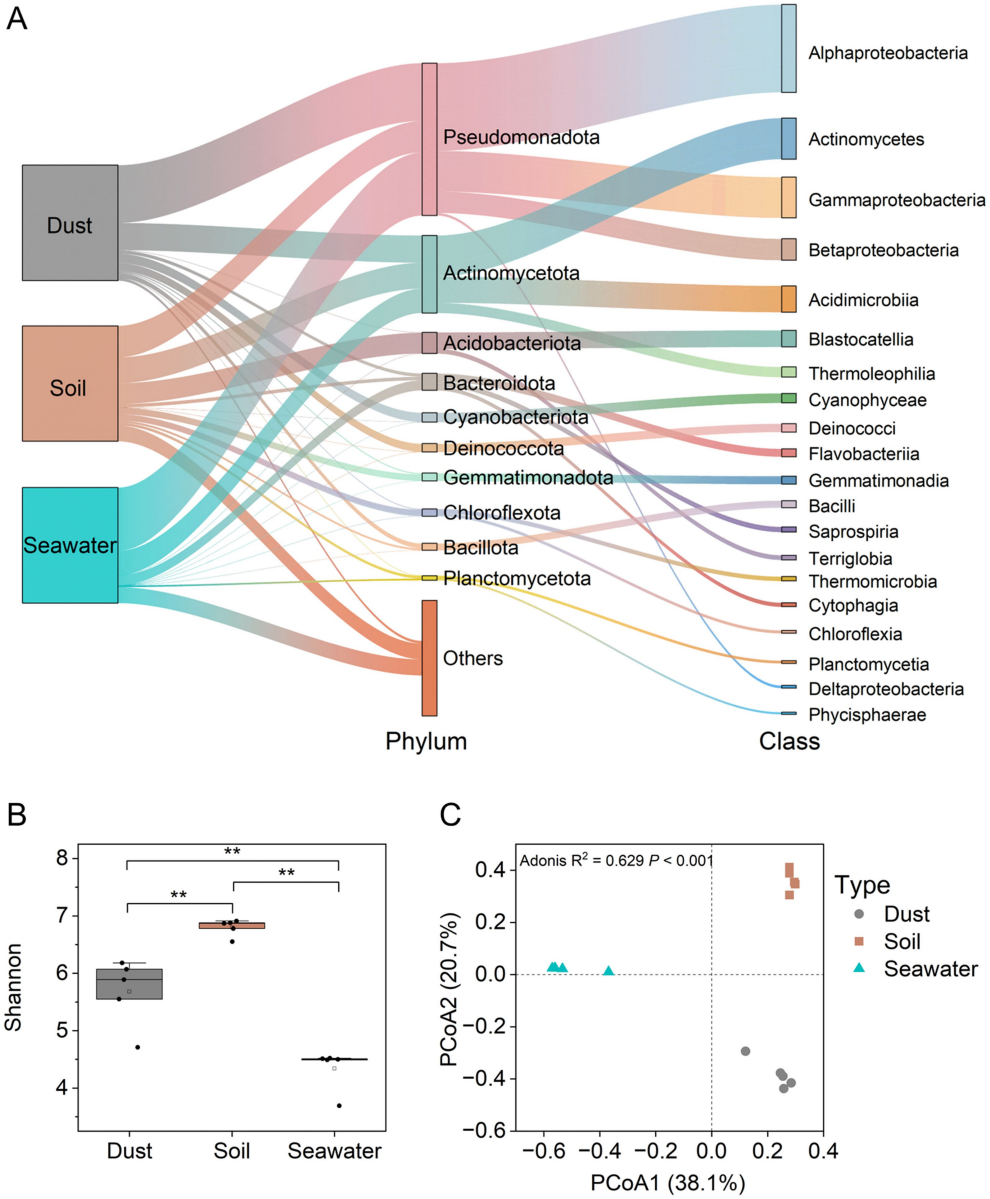
Taxonomic compositions and diversity of bacterial communities in dust, soil, and seawater samples. Taxonomic compositions of the bacterial communities **(A)**. Alpha diversity (Shannon index) of the bacterial communities **(B)**. The Wilcoxon rank-sum test was used to compare the differences in Shannon indices of the bacterial communities among dust, soil, and seawater samples (*p < 0.05, **p < 0.01, ***p < 0.001). Beta diversity (principal coordinate analysis (PCoA) based on the Bray–Curtis distance) of bacterial communities **(C)**.

Using 16S rRNA gene sequencing, the bacterial community structures in dust, soil, and seawater samples were characterized with an average of 63,533 high-quality sequences per sample. All of the rarefaction curves approached the plateau phase (Supplementary Figure 1), indicating that the diversity was adequately captured by the sequenced reads across different samples. Pseudomonadota (27.2–54.7%) and Actinomycetota (18.2–23.6%) were the dominant phyla across all three sample types (Figure 2A). Notably, Cyanobacteriota and Deinococcota constituted significant proportions of the bacterial communities in dust samples. The bacterial taxonomic structure at the class level varied among the different sample types, with Alphaproteobacteria (40.2%) being predominant in dust, Actinomycetes (14.1%) being predominant in soil, and Gammaproteobacteria (27.0%) being predominant in seawater. At the class level, the abundance of Flavobacteriaceae was significantly (*p* < 0.05) positively correlated with the proportion of As(III) but negatively correlated with the proportion of As(V) in dust and soil (Supplementary Figure 2). The bacterial alpha diversity index (Shannon index) significantly differed (*p* < 0.05) among the sample types, with soil exhibiting greater diversity than dust or seawater (Figure 2B). Furthermore, bacterial beta diversity significantly varied among dust, soil, and seawater samples (Bray-Curtis PCoA, Adonis test; *p* < 0.001; *R*^2^ = 0.629; Figure 2C). Specifically, the bacterial communities in the seawater samples were distinct from those in the dust and soil samples along the first principal coordinate axis (PCo1), whereas those in the dust and soil samples were further differentiated along the second principal coordinate axis (PCo2).

**Figure 3:**
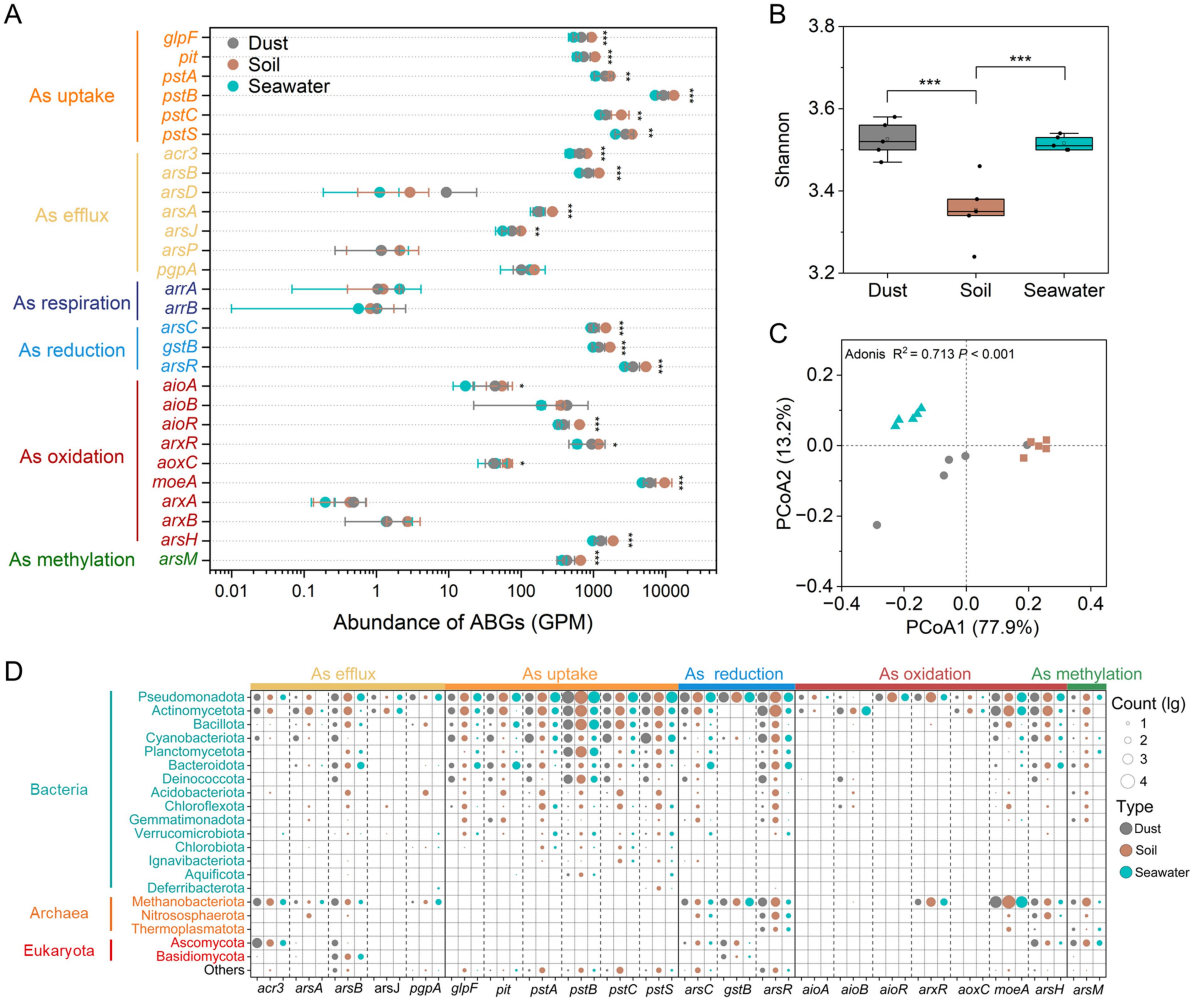
Abundance, diversity, and taxonomic compositions of arsenic biotransformation genes (ABGs). The abundances (genes per million, GPM) of ABGs **(A)**. Alpha diversity (Shannon index) of ABGs **(B)**. The Wilcoxon rank-sum test was used to compare the differences in abundances and Shannon indices of ABGs among dust, soil, and seawater samples (*p < 0.05, **p < 0.01, ***p < 0.001). Beta diversity (principal coordinate analysis (PCoA) based on the Bray–Curtis distance) of ABGs **(C)**. Taxonomic compositions of ABGs at the phylum level **(D)**.

Using metagenomic sequencing, 28 ABGs were identified across dust, soil, and seawater samples (Figure 3A). The regulatory gene (*arsR*) presented the highest abundance among the *ars* genes in dust (3530.02 ± 789.92 GPM), soil (5317.66 ± 459.04 GPM), and seawater (2697.69 ± 193.92 GPM). Significant positive correlations (*p* < 0.001) were observed among the abundances of different categories of ABGs, except for arsenic respiration (Supplementary Figure 3). The abundance of the *arsC* gene exhibited a significantly positive correlation (*p* < 0.01) with the proportion of As(III) in dust and soil, whereas the abundance of the *arsM* gene showed no significant correlation (*p* > 0.05) with the proportion of methylated arsenic in dust and soil (Supplementary Figure 4). Overall, the abundance of most ABGs varied across different sample types in the order soil > dust > seawater. However, the Shannon indices of the ABGs were significantly (*p* < 0.05) lower in the soil samples than in the dust and seawater samples (Figure 3B). The beta diversity of the ABGs also significantly varied among the dust, soil, and seawater samples (Bray-Curtis PCoA, Adonis test; *p* < 0.001; *R*^2^ = 0.713; Figure 3C). Specifically, the soil and seawater samples each produced two clearly separated clusters, whereas the dust samples formed one mildly dispersed cluster. Furthermore, 92.76%, 5.57%, and 1.66% of the ABG sequences were classified as bacteria, archaea, and eukaryota, respectively (Figure 3D). Pseudomonadota, Methanobacteriota, and Ascomycota emerged as the most dominant bacterial, archaeal, and eukaryotic carriers of ABGs, respectively. Notably, Cyanobacteriota were significantly more important contributors to ABGs in dust than in soil and seawater. In terms of the distribution of ABGs in microorganisms, ABGs, including the *acr3*, *arsB*, *arsC*, *gstB*, *arsH*, and *arsM* genes, were widely distributed across bacteria, archaea, and eukaryotes.

### 3.3 Bacterial and ABG source tracking

**Figure 4:**
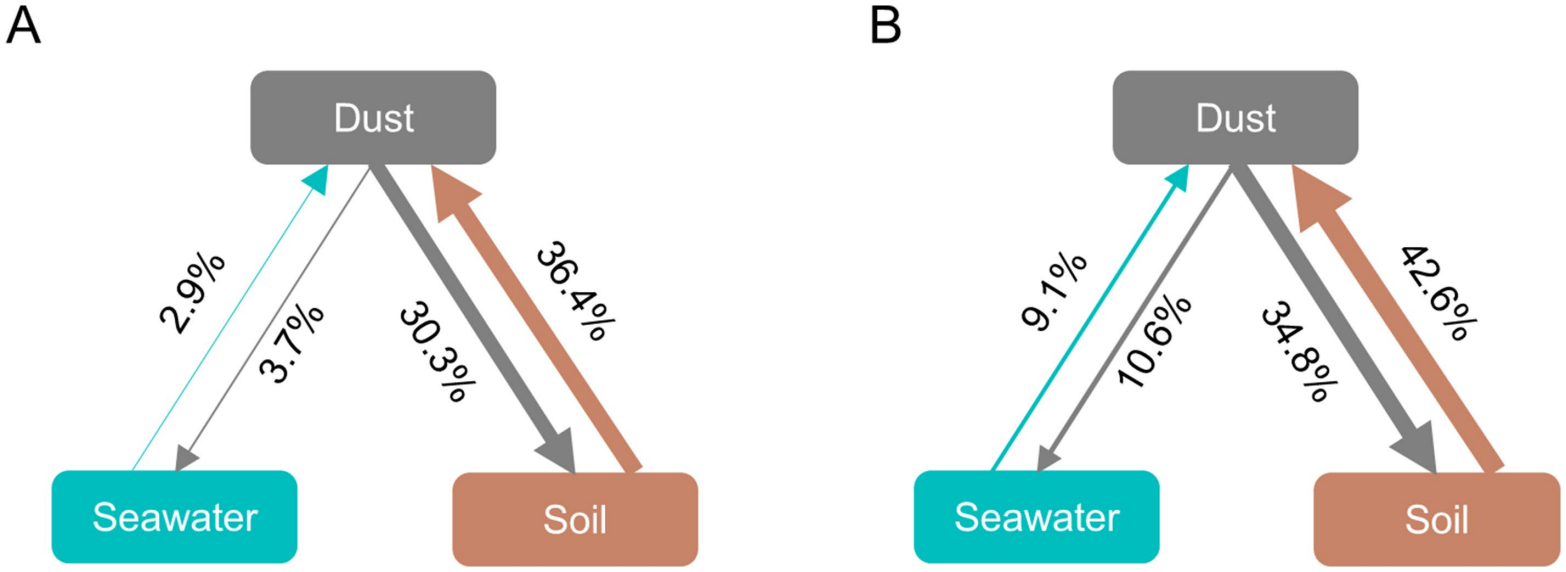
Fast expectation–maximization microbial source tracking (FEAST) analysis for bacteria **(A)** and ABGs **(B)**. The direction of the arrows indicates the relationship between sources and sinks, and the percentages reflect the contribution of each source.

Since dust, soil, and seawater may achieve a certain degree of balance after coexisting for long periods at the same site, FEAST analysis was employed to assess their mutual contribution rates for bacteria and ABGs by assuming dust to be a ‘sink’ or a ‘source’ (Figures 4A,B). When dust was assumed to be the ‘sink’, approximately 36.4% of the dust-associated bacteria were sourced from soil, and 2.9% were sourced from seawater. Similarly, 42.6% of the ABGs in dust originated from soil, and 9.1% originated from seawater. When dust was considered the ‘source’, approximately 30.3% and 3.7% of the bacterial communities in soil and seawater, respectively, along with 34.8% and 10.6% of the ABGs in soil and seawater, respectively, originated from dust.

### 3.4 Key bacterial taxa and ABGs

**Figure 5:**
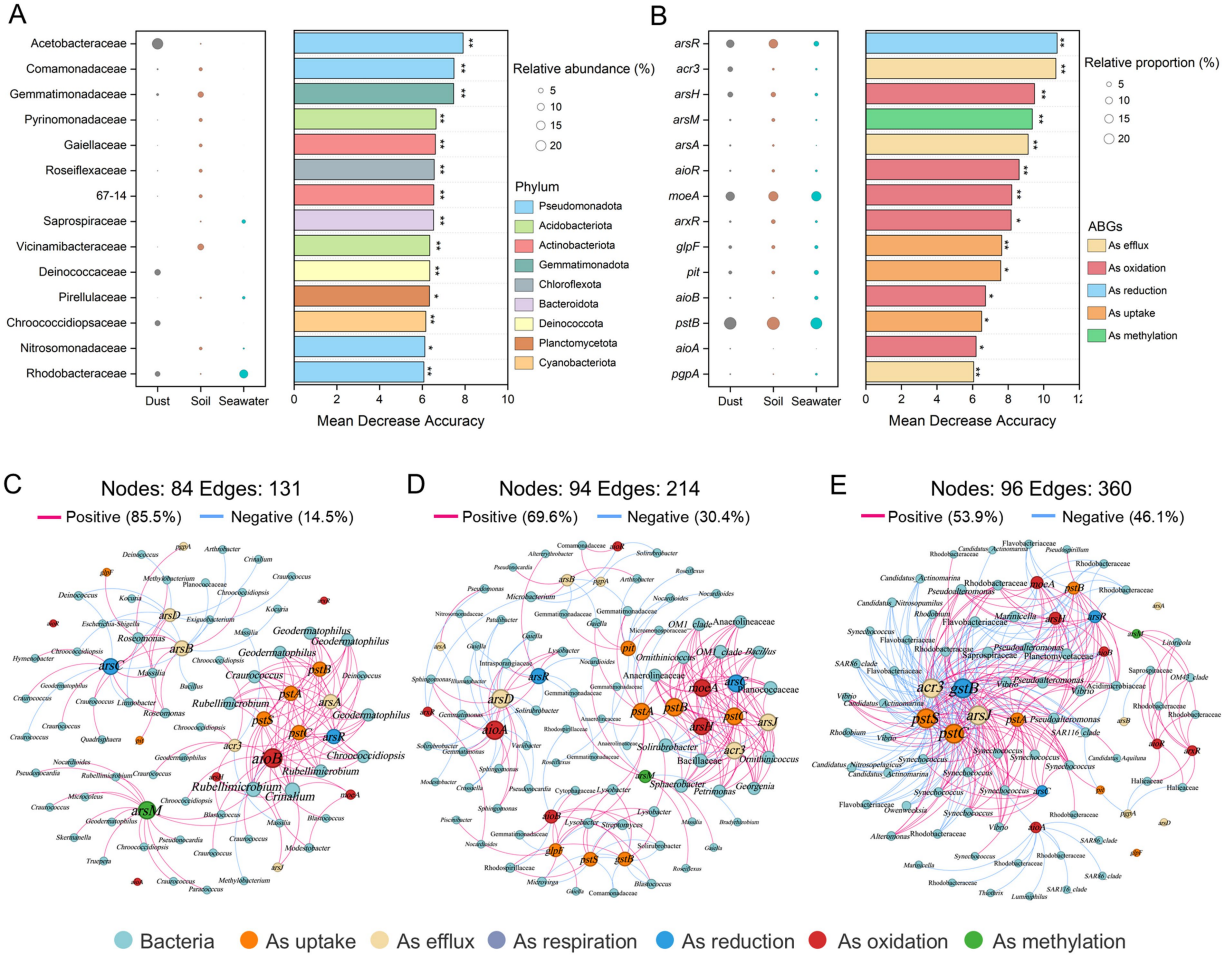
Key bacterial taxa and arsenic biotransformation genes (ABGs) and the co-occurrence networks of bacterial taxa and ABGs across dust, soil, and seawater. Random forest classification models identifying key bacterial taxa at the family level **(A)** and ABGs **(B)**. The panels on the left illustrate the relative abundance of these families or the relative proportions of ABGs. The panels on the right show the mean decrease in accuracy for each family or ABG, indicating their importance in the model (*p < 0.05 and **p < 0.01). Network analysis revealing the co-occurrence patterns between ABGs and bacterial communities in dust **(C)**, soil **(D)**, and seawater **(E)** based on Spearman correlation analysis (|r| > 0.50, *p* < 0.05).

Key bacterial taxa were identified using a random forest classification model and ranked in descending order of their importance (Figure 5A). Among these key bacterial taxa, the Acetobacteraceae (Pseudomonadota), Deinococcaceae (Deinococcota), and Chroococcidiopsidaceae (Cyanobacteriota) presented significantly greater relative abundances (*p* < 0.001) in dust than in soil and seawater. Furthermore, network analysis was conducted to identify the highly connected taxa within the bacterial community. The bacterial co-occurrence networks in dust, soil, and seawater all displayed scale-free and small-world characteristics (Supplementary Table 3; small-world coefficient (*σ*) *>* 1). Compared with those in dust and seawater, the bacterial networks in soil presented a greater number of nodes and a lower proportion of positive correlations (Supplementary Figure 5). A total of one module hub and three connector hubs were identified in the dust network (Supplementary Figure 6). These hubs, which represented keystone taxa, belonged to Acetobacteraceae, Deinococcaceae, and Rhodobacteraceae (Supplementary Table 4).

Fourteen key ABGs were also identified via a random forest classification model (Figure 5B). Among these key ABGs, the relative proportion of the *acr3* gene, which is involved in arsenic efflux, was significantly greater (*p* < 0.001) in dust (4.78%) than in soil (0.89%) or seawater (0.86%). Furthermore, network analysis revealed significant co-occurrence relationships between ABGs and bacterial communities in dust, soil, and seawater, with the *arsR*, *arsC, acr3*, and *pstSCAB* genes emerging as critical nodes (Figures 5C–E). The *aioB* and *arsM* genes were also identified as key nodes closely associated with bacterial communities in the co-occurrence network of dust. *Rubellimicrobium* (belonging to Roseobacteraceae), *Geodermatophilus* (belonging to Geodermatophilaceae), and *Craurococcus* (belonging to Acetobacteraceae) were closely associated with ABGs in the dust network.

**Figure 6:**
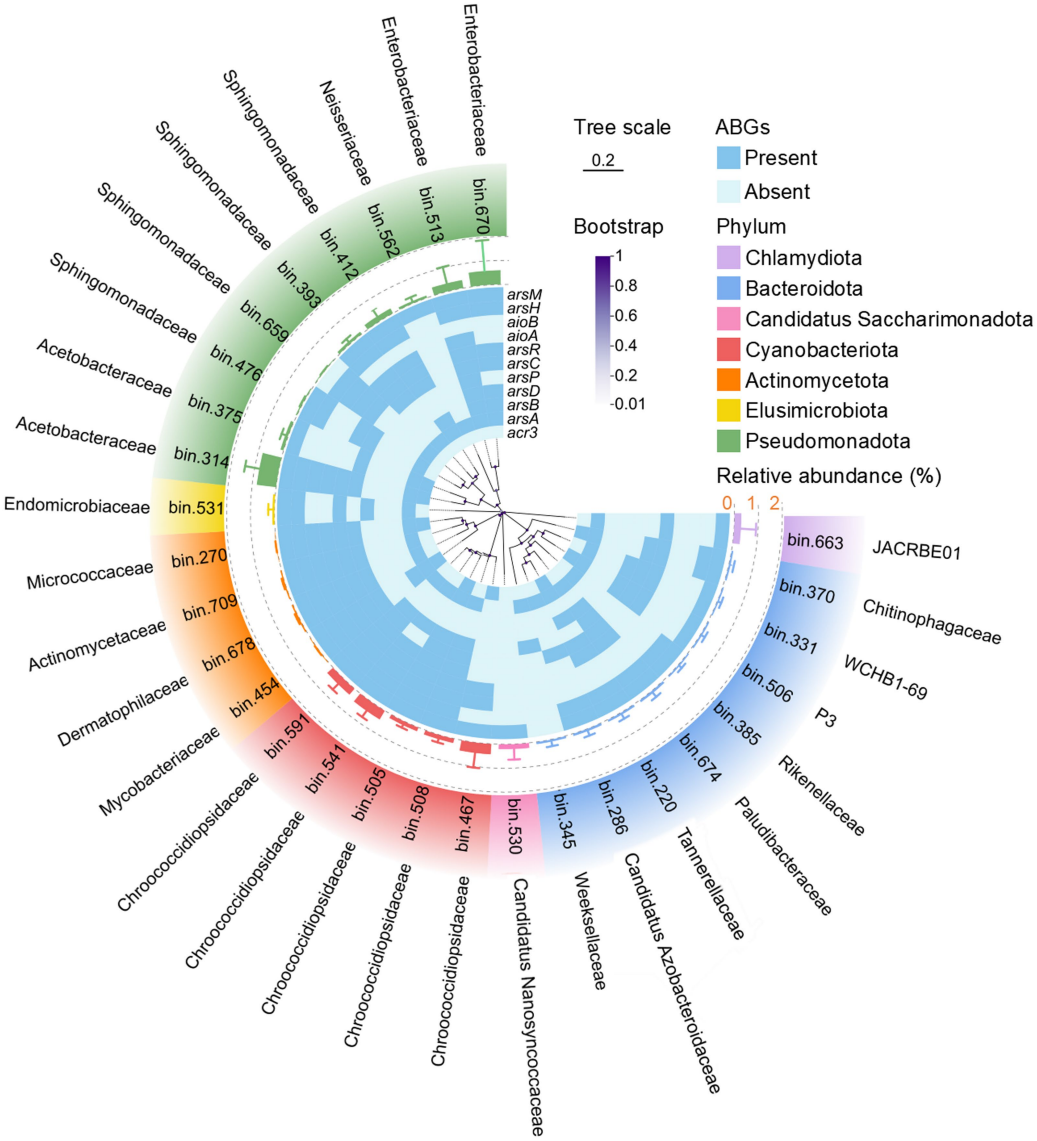
Phylogenetic tree of the detected bacterial metagenome-assembled genomes (MAGs) with completeness greater than 80% and contamination less than 10% in dust. The line bar indicates a tree scale of 0.2, and the purple dots represent bootstrap values. The heatmap illustrates the presence (blue) or absence (light blue) of arsenic biotransformation genes (ABGs) for each MAG. The bars enclosed by dashed rings depict the relative abundance of each MAG in dust.

Furthermore, at the genome level, 32, 18, and 35 MAGs with completeness above 80% and contamination below 10% were recovered from dust, soil, and seawater, respectively (Supplementary Figure 7). The predominant phyla for bacterial and archaeal MAGs were Pseudomonadota and Nitrososphaerota, respectively. The relative abundance and presence/absence of ABGs were further analyzed for each MAG in dust (Figure 6; Supplementary Figure 8). The MAG related to Acetobacteraceae (Bin 314) presented the highest relative abundance in dust. Additionally, Chroococcidiopsidaceae MAGs (Bins 467, 541, and 591) presented high relative abundances in dust. Furthermore, although a diverse array of ABGs was observed across different MAGs, a majority of these high-abundance MAGs in dust harbored key ABGs, including the *arsR*, *arsC*, *aioA*, *aioB*, and *arsM* genes.

## 4 Discussion

In this study, dust samples from Xiamen, China, presented significantly higher (*p* < 0.05) total arsenic concentrations than soil samples did, which is consistent with findings from Nevada, United States (Soukup et al., 2012), and Ahvaz, Iran (Ghanavati et al., 2018). The elevated arsenic levels in dust may be attributed to the fact that dust particles are generally smaller in size and more susceptible to contamination from anthropogenic activities than soil particles are (Soukup et al., 2012). Additionally, the higher CV values for arsenic in the dust samples further suggest that arsenic concentrations in dust are more substantially influenced by variations attributable to diverse anthropogenic activities (Bullard and Baddock, 2019; Chen et al., 2018a). Inorganic arsenic species dominated in both dust and soil samples, but methylated arsenic species were also detected. The detection of MAs(V) and DMAs(V) in soils is possible because of microorganism-mediated biomethylation processes, whereas the occurrence of TMAs(V)O in dust likely results from the atmospheric oxidation of volatile trimethylarsine (TMAs) emitted from terrestrial and marine environments (Qiao et al., 2023; Savage et al., 2019; Yan et al., 2024; Zhang et al., 2015; Zhao et al., 2013).

The diversity of both the bacterial communities and the ABGs significantly differed (*p* < 0.05) among the dust, soil, and seawater samples. The Shannon indices of bacterial communities were significantly (*p* < 0.05) greater in soil than in dust and seawater, which is consistent with the recognized role of soil as one of the most extensive reservoirs of biological diversity on Earth (Swift et al., 1998). Furthermore, the majority of ABGs showed significantly (*p* < 0.05) greater abundances in soil than in dust and seawater, possibly because soil can provide a nutrient-rich medium that supports diverse microbial activities (Yadav et al., 2021). However, the Shannon indices of ABGs were significantly greater (*p* < 0.05) in dust and seawater than in soil. This observation may be attributed to the harsher environmental conditions in both dust and seawater, with limited nutrient availability likely constraining microbial diversity while promoting gene diversity (Fierer et al., 2012). Compared with those in soil, bacterial networks in dust and seawater presented fewer nodes and higher proportions of positive correlations, further suggesting that bacterial communities exhibit more cooperative relationships when subjected to harsh environmental conditions such as those in dust and seawater (Li et al., 2022). Notably, bacterial beta diversity analysis revealed that bacterial communities in dust are more closely related to those in soil than to those in seawater, which was supported by the greater contribution of soil compared with seawater as a source of bacteria in dust. Additionally, in the PCoA scatter plots visualizing the beta diversity of the ABGs, the dust samples displayed a more dispersed pattern than the soil and seawater samples did, indicating considerable spatial variability in the profiles of the ABGs among the dust samples (Yan et al., 2024).

The source-sink relationships of both bacteria and ABGs were investigated using the FEAST analysis. When dust is considered the ‘sink’, the great contributions of soil to both bacteria and ABGs in dust indicate that soil serves as the dominant source for dust-associated microorganisms. Consistent with these results, previous studies have reported that terrestrial bacterial sources contribute significantly to airborne and dust-associated bacteria (Qi et al., 2023; Salawu-Rotimi et al., 2021). Notably, soil exhibited a greater contribution rate for ABGs than for bacteria in dust. A previous study reported that fungal communities, rather than bacterial communities, in dust were more strongly associated with soil microbiomes since bacteria experience more selective fractionation during the generation of dust (Salawu-Rotimi et al., 2021). Therefore, given that ABGs are associated with both prokaryotic and eukaryotic microorganisms, it is unsurprising that soil significantly contributes to the abundance of ABGs hosted by both bacteria and fungi in dust. Additionally, the arsenic in dust has been reported to originate from terrestrial sources (Ma et al., 2021; Yan et al., 2024). Consequently, soil could not only be a significant source of arsenic but also serve as a considerable contributor to the associated genes in dust. Additionally, although the contribution rate was not high, seawater could also contribute to the presence of some specific bacteria in dust. For instance, Cyanobacteriota was detected as an important bacterial community in dust samples in this study. A previous study reported that the presence of Cyanobacteriota adhering to atmospheric particulate matter is likely attributable to sea and lake spray aerosols, which facilitate the transport of Cyanobacteriota from aquatic environments into the atmosphere (Nie et al., 2023). Both soil and seawater in Tokyo Bay were also identified as consistent and predominant sources of airborne microorganisms in Tokyo, Japan (Uetake et al., 2019). When dust was considered the ‘source’, the contribution of dust to both bacterial communities and ABGs in soil was more significant than that in seawater. Intriguingly, the contribution rates for bacteria and ABGs from dust to seawater were greater than those from seawater to dust, indicating that dust predominantly serves as a primary source of both bacteria and ABGs in seawater. Previous studies have shown that a variety of microorganisms can be transported via dust and aerosol dispersal, influencing marine microbial communities and biogeochemical processes (Berman-Frank and Rahav, 2012; Rahav et al., 2016).

Pseudomonadota were the dominant bacterial phylum according to 16S rRNA gene sequencing and predominated among MAGs recovered through metagenomic binning across dust, soil, and seawater samples, which is consistent with the well-known prevalence of Pseudomonadota in diverse environments (Li et al., 2022; Suh et al., 2015; Wang et al., 2019; Xu et al., 2014). Furthermore, the ubiquitous Pseudomonadota were also the dominant bacterial carriers of ABGs, emphasizing their importance in arsenic biotransformation (Saunders et al., 2019; Zhang et al., 2015). Cyanobacteriota, often identified in bioaerosols (Hu et al., 2020; Liu et al., 2018; Wei et al., 2020), accounted for relatively high proportions of bacterial communities in dust samples. The MAGs affiliated with Cyanobacteriota also presented high relative abundances in dust samples, which is consistent with their large proportions in bacterial communities in dust. Deinococcota also constituted a significant component of bacterial communities in dust, as certain genera within this phylum exhibit remarkable resistance to solar radiation (Xu et al., 2017). Furthermore, according to the random forest analysis, Acetobacteraceae, belonging to Pseudomonadota, Deinococcaceae, belonging to Deinococcota, and Chroococcidiopsidaceae, belonging to Cyanobacteriota, which exhibited high abundances in dust samples, were identified as key taxa. Notably, the keystone taxa within the bacterial co-occurrence network predominantly belonged to Acetobacteraceae and Deinococcaceae. Therefore, Acetobacteraceae and Deinococcaceae not only serve as key taxa characterized by high abundances in dust but also function as keystone taxa with high connectivity within bacterial communities in dust environments. Furthermore, the Acetobacteraceae MAGs (Bin 314) and Chroococcidiopsidaceae MAGs (Bins 467, 541, and 591) presented high relative abundances in dust samples. These two bacterial families are commonly found in extreme environments, such as deserts, underscoring their critical role in adaptation to soil conditions with limited water (Al Ashhab et al., 2021; Caiola and Billi, 2007; Yang et al., 2017). Consequently, they were unsurprisingly key microbial constituents in dust, which can also be considered an arid environment.

Across dust, soil, and seawater samples, highly abundant ABGs involved in arsenic uptake (*pstSCAB*) (Rosen, 2002), oxidation (*moeA* and *arsH*) (Zhu et al., 2017; Chen et al., 2015), reduction (*arsC* and *gstB*) (Chrysostomou et al., 2015), As(III) efflux (*arsB* and *acr3*) (Garbinski et al., 2019), and methylation (*arsM*) (Qin et al., 2006) were detected. Additionally, these ABG categories exhibited significant positive correlations with each other (*p* < 0.001). These findings indicate that multiple arsenic transformation processes commonly coexist within atmospheric, terrestrial, and aquatic environments (Zhu et al., 2017). Additionally, the wide distribution of the *acr3*, *arsB*, *arsC*, *gstB*, *arsH*, and *arsM* genes across bacteria, archaea, and eukaryotes aligns with previous studies reporting their broad presence in prokaryotes and eukaryotes (Chen et al., 2020; Li et al., 2024). Notably, the *arrAB* gene, which is responsible for As(V) respiration, was detected at extremely low levels in dust, which can be attributed to the prolonged exposure of dust to abundant oxygen in the air (Min et al., 2022; Yan et al., 2024). According to the random forest analysis, the *acr3* gene, which presented a significantly greater relative proportion (*p* < 0.001) in dust samples than in soil and seawater samples, was identified as one of the key ABGs. Therefore, arsenic efflux, widely acknowledged as an effective arsenic detoxification mechanism (Castro-Severyn et al., 2022), plays a pivotal role in arsenic biotransformation and resistance, particularly in dust samples with relatively high arsenic concentrations. Moreover, a significantly positive correlation (*p* < 0.01) was observed between the abundance of the *arsC* gene and the proportion of As(III) in dust and soil. This observation is expected since the *arsC* gene was the key gene involving in As(V) reduction to As(III) (Zhu et al., 2017). Furthermore, the abundance of Flavobacteriaceae exhibited a significantly (*p* < 0.05) positive correlation with the proportion of As(III) and a negative correlation with the proportion of As(V) in dust and soil. This suggests that Flavobacteriaceae may play an important role in arsenic reduction in the environment. Supporting this, a novel species within the family Flavobacteriaceae was identified as a multi-metal-resistant bacterium harboring the *arsC* gene (Li S. et al., 2023). However, no significant correlation (*p* > 0.05) was observed between the abundance of the *arsM* gene and the proportion of methylated arsenic in dust and soil. Similar findings have been also reported in previous studies, which may be attributed to the influence of other arsenic biotransformation pathways (e.g., arsenic demethylation) and chemical processes on the proportion of methylated arsenic in the environment (Qiao et al., 2023; Yan et al., 2024).

Co-occurrence networks between bacterial taxa and ABGs, together with MAG binning, were utilized to explore the relationships between bacteria and ABGs. *Craurococcus*, a genus within Acetobacteraceae, was closely associated with a variety of ABGs, including *acr3*, *arsA*, *arsC*, and *arsM* genes in the co-occurrence network between bacterial taxa and ABGs in dust. Furthermore, Acetobacteraceae MAGs were also found to harbor these ABGs. These findings further suggest that Acetobacteraceae are likely important hosts for ABGs within dust environments (Cernava et al., 2018). The *arsR*, *arsC*, *acr3*, and *pstSCAB* genes represented key nodes in the co-occurrence networks linking ABGs and bacterial communities across dust, soil, and seawater. This finding indicates that after uptake via the PstSCAB system, As(V) can be reduced to As(III) by ArsC and subsequently effluxed by Acr3, thereby forming an essential arsenic detoxification pathway (Garbinski et al., 2019). Furthermore, the *arsR*, *arsC*, *acr3, aioB*, and *arsM* genes were not only identified as key nodes closely associated with bacterial communities in the co-occurrence network of dust but also harbored by the majority of high-abundance MAGs in dust. These results indicate that dust may possess the capacity for multiple types of arsenic biotransformation, including As(V) reduction, As(III) oxidation, and As(III) methylation (Yan et al., 2024).

## 5 Conclusion

This study revealed that the bacterial community structures and ABG profiles in dust were significantly different from those in soil and seawater. Soil, rather than seawater, was the main source of both bacteria and ABGs in dust. A variety of bacteria adapted to arid environments, particularly Acetobacteraceae showed high abundance and network connectivity in dust and was identified as a key taxon according to the random forest. The MAGs with high relative abundance in dust were also affiliated with Acetobacteraceae and harbored several key ABGs. Acetobacteraceae could be a potentially important contributor to arsenic biotransformation in dust. Furthermore, ABGs, particularly the *arsR*, *arsC*, *acr3, aioB*, and *arsM* genes, exhibited close connections with bacterial taxa in the co-occurrence networks between ABGs and bacterial communities in dust and were prevalent within the high-abundance MAGs in dust. Further research is essential to comprehensively investigate the capacity of arsenic biotransformation within dust.

## Data Availability

The 16S rRNA gene sequencing data and metagenomic sequencing data have been deposited in the NCBI SRA under BioProject PRJNA1214267 and PRJNA1214535, respectively.

## Footnotes

^1^ https://github.com/XinweiSong/Asgene
