# Supplementary figures and images for: Distinct bacterial community structures and arsenic biotransformation gene profiles in dust
Source: Front Microbiol. 2025 Jul 30;16:1607082. doi: 10.3389/fmicb.2025.1607082 (PMC12343739; doi:10.3389/fmicb.2025.1607082)

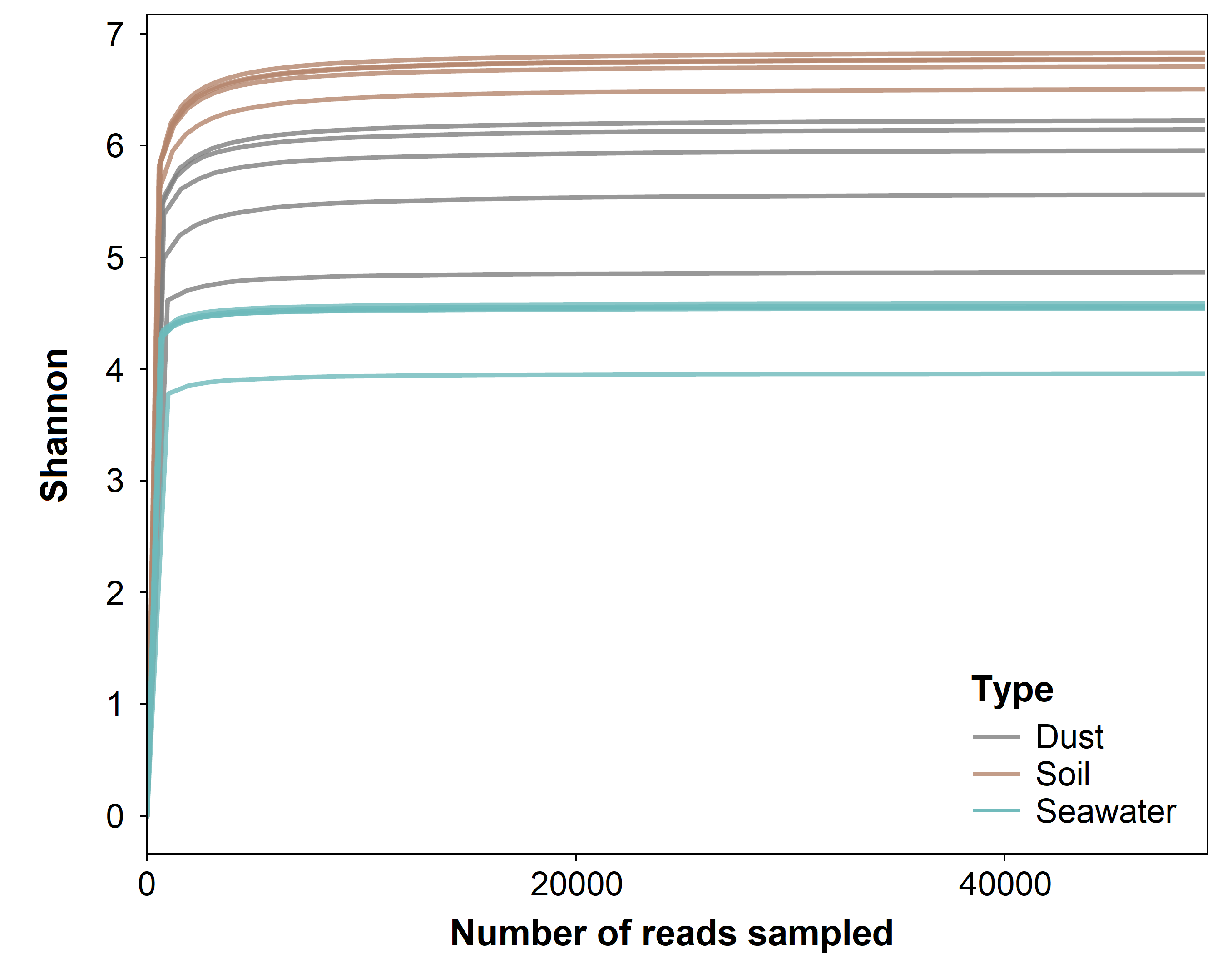

Supplement: Supplementary file 1 [file Image_1.tif]

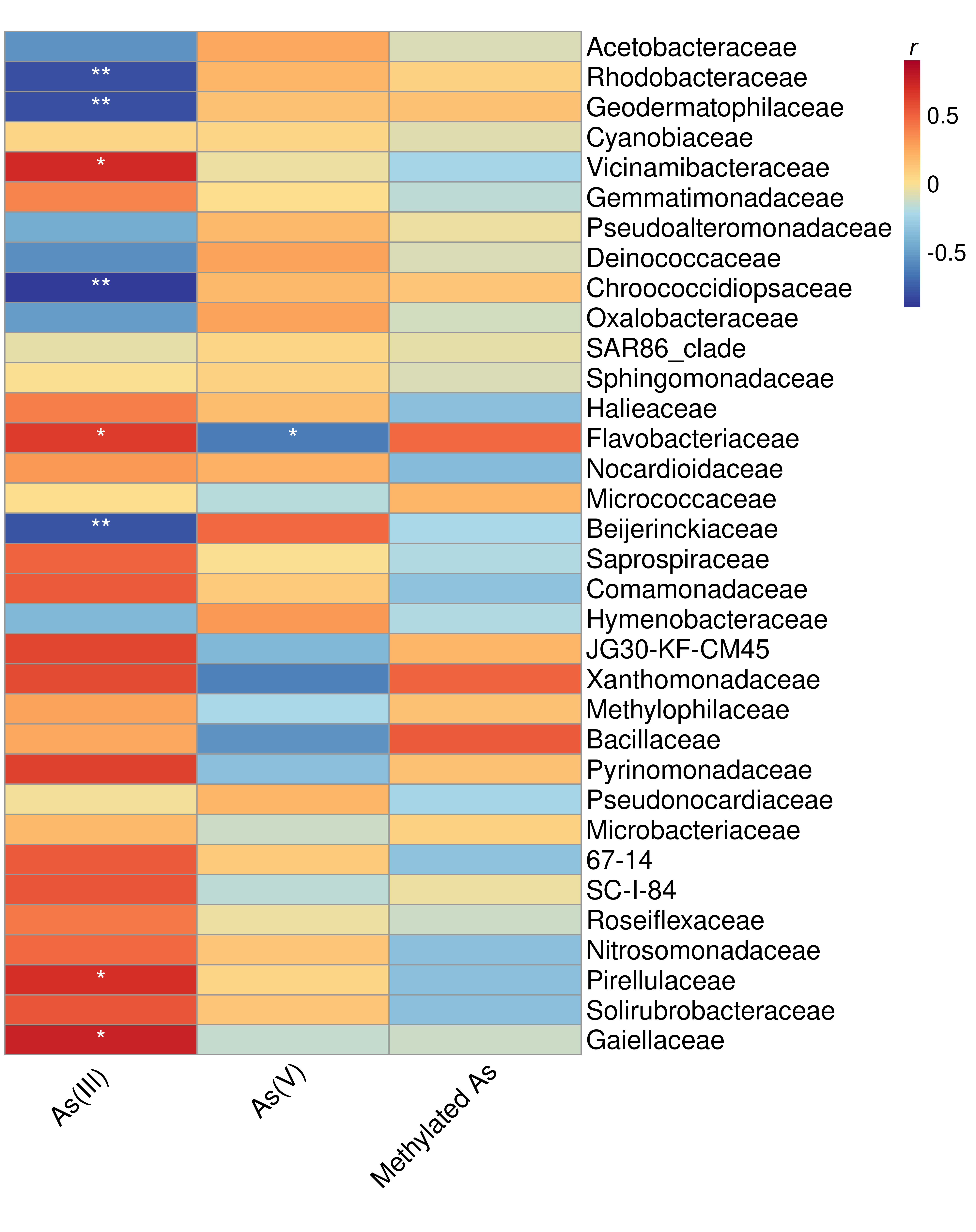

Supplement: Supplementary file 2 [file Image_2.tif]

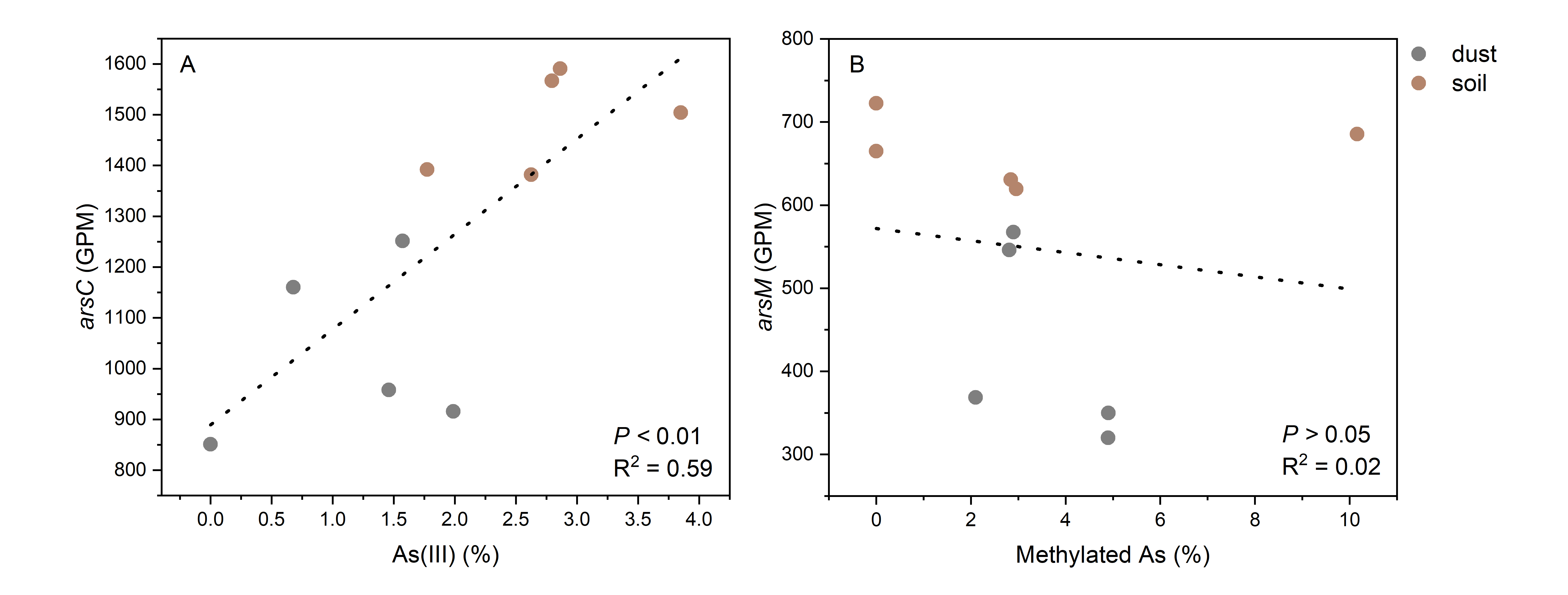

Supplement: Supplementary file 4 [file Image_4.tif]

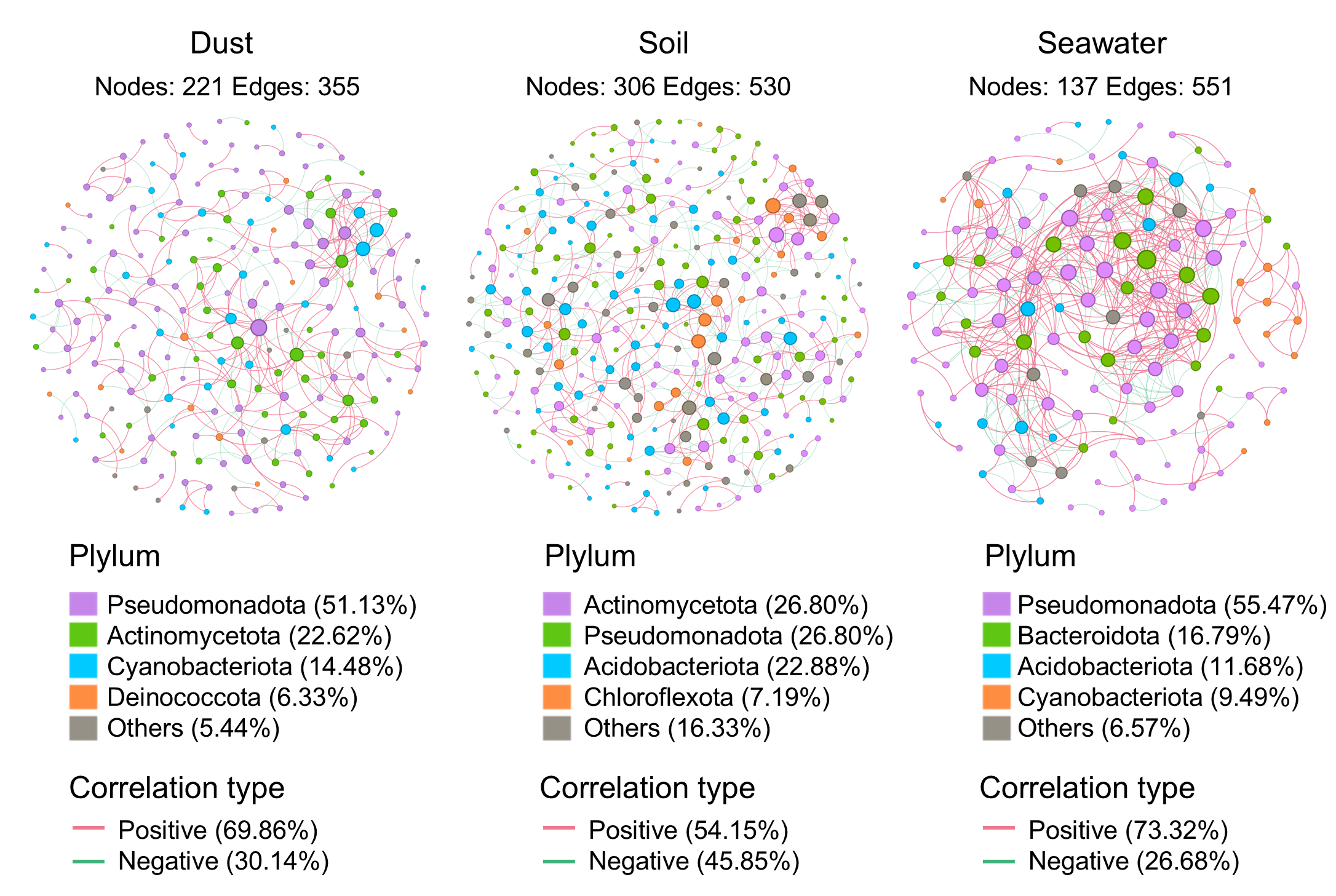

Supplement: Supplementary file 5 [file Image_5.tif]

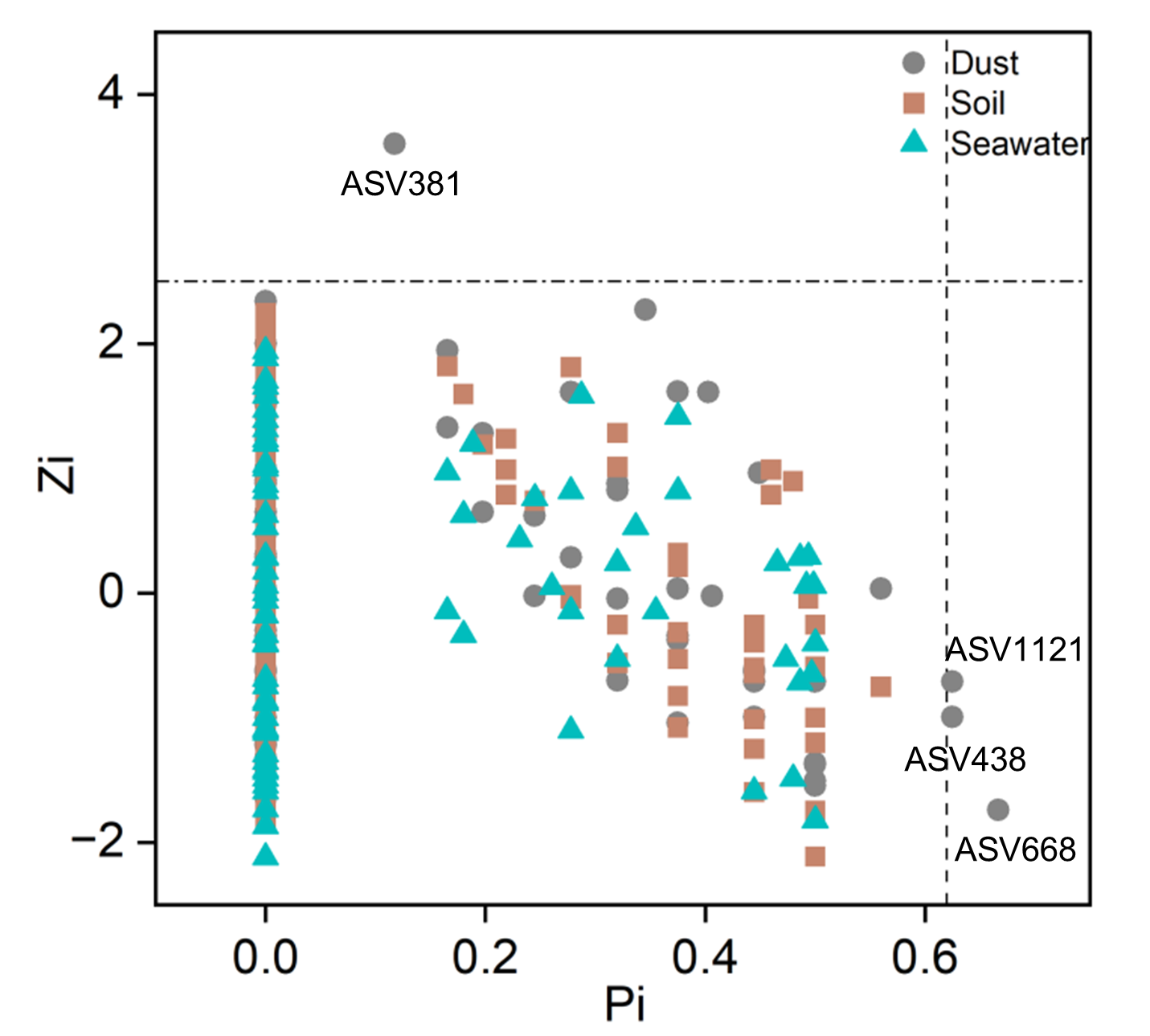

Supplement: Supplementary file 6 [file Image_6.tif]

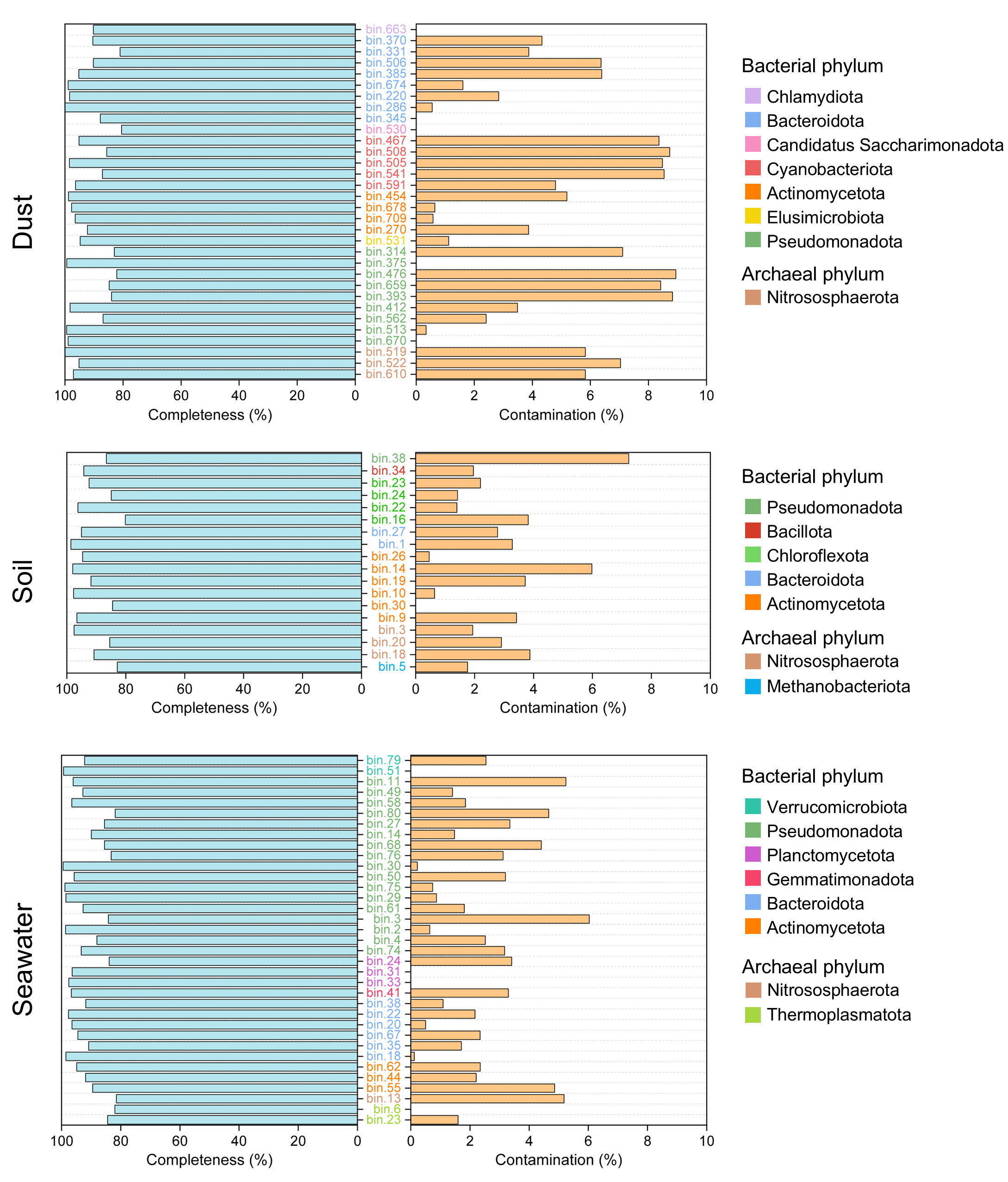

Supplement: Supplementary file 7 [file Image_7.tif]
