# Supplementary material for: Distinct bacterial community structures and arsenic biotransformation gene profiles in dust
Source: Front Microbiol. 2025 Jul 30;16:1607082. doi: 10.3389/fmicb.2025.1607082 (PMC12343739; doi:10.3389/fmicb.2025.1607082)
Supplement: Supplementary file 8 [file Table_1.docx]

**Supplementary Table 1.** Total arsenic concentrations in dust, soil, and seawater.

| Type | Unit | Max | Min | Mean | SD | CV (%) |
| --- | --- | --- | --- | --- | --- | --- |
| Dust (n = 5) | mg⋅kg^−1^ | 57.18 | 8.49 | 19.75 | 19.25 | 97.48 |
| Soil (n = 5) | mg⋅kg^−1^ | 10.41 | 3.18 | 5.95 | 2.26 | 37.99 |
| Seawater (n = 5) | μg⋅L^−1^ | 2.49 | 1.50 | 1.79 | 0.31 | 17.43 |

Notes: SD: Standard deviation; CV: Coefficient of variation.
