# Supplementary material for: Distinct bacterial community structures and arsenic biotransformation gene profiles in dust
Source: Front Microbiol. 2025 Jul 30;16:1607082. doi: 10.3389/fmicb.2025.1607082 (PMC12343739; doi:10.3389/fmicb.2025.1607082)
Supplement: Supplementary file 9 [file Table_2.docx]

**Supplementary Table 2.** Proportions (%) of arsenic species in dust and soil.

| Arsenic species (%) | Dust (n = 5) | Soil (n = 5) |
| --- | --- | --- |
| As(V) | 95.07 ± 1.55 | 94.02 ± 4.64 |
| As(III) | 1.43 ± 0.47 | 2.78 ± 0.73 |
| MAs(V) | 0.59 ± 0.92 | 1.93 ± 2.17 |
| DMAs(V) | 0.38 ± 0.48 | 1.27 ± 2.16 |
| TMAs(V)O | 2.53 ± 1.61 | N.D. |

Notes: N.D: Not detected.
