# Supplementary material for: Distinct bacterial community structures and arsenic biotransformation gene profiles in dust
Source: Front Microbiol. 2025 Jul 30;16:1607082. doi: 10.3389/fmicb.2025.1607082 (PMC12343739; doi:10.3389/fmicb.2025.1607082)
Supplement: Supplementary file 10 [file Table_3.docx]

**Supplementary Table 3.** Topological properties of co-occurrence networks and their identically sized random networks of bacterial communities under different sample types.

| Type |  | Empirical network | | | | | |  | Random network | | |
| --- | --- | --- | --- | --- | --- | --- | --- | --- | --- | --- | --- |
|  |  | Node | Edge | CC | APL | MD | σ |  | CC_r_ | APL_r_ | MD_r_ |
| Dust |  | 221 | 355 | 0.315 | 5.167 | 0.791 | 14.838 |  | 0.018 ± 0.007 | 4.381 ± 0.090 | 0.573 ± 0.009 |
| Soil |  | 306 | 530 | 0.363 | 10.231 | 0.839 | 9.526 |  | 0.016 ± 0.005 | 4.296 ± 0.052 | 0.522 ± 0.007 |
| Seawater |  | 137 | 551 | 0.540 | 3.454 | 0.551 | 4.043 |  | 0.105 ± 0.011 | 2.715 ± 0.037 | 0.275 ± 0.007 |

Notes: CC, average clustering coefficient; APL, average shortest path length; MD, modularity; σ, small-world coefficient σ = (CC/CC_r_)/(APL/APL_r_) and σ *>* 1 indicates ‘small-world’ properties, namely, high interconnectivity and high efficiency. The subscript r indicates the properties of the random network. The average values and standard deviations of CC_r_, APL_r_, and MD_r_ of the 100 random networks were calculated.
