# Supplementary material for: Distinct bacterial community structures and arsenic biotransformation gene profiles in dust
Source: Front Microbiol. 2025 Jul 30;16:1607082. doi: 10.3389/fmicb.2025.1607082 (PMC12343739; doi:10.3389/fmicb.2025.1607082)
Supplement: Supplementary file 11 [file Table_4.docx]

**Supplementary Table 4.** Network roles and corresponding taxonomic classifications (phylum, class, order, and family) of keystone taxa within the bacterial co-occurrence network.

| ID | ASV381 | ASV438 | ASV668 | ASV1121 |
| --- | --- | --- | --- | --- |
| Sample type | Dust | Dust | Dust | Dust |
| Module | 3 | 1 | 4 | 3 |
| Node Type | Module hubs | Connector hubs | Connector hubs | Connector hubs |
| Phylum | Pseudomonadota | Deinococcota | Pseudomonadota | Pseudomonadota |
| Class | Alphaproteobacteria | Deinococci | Alphaproteobacteria | Alphaproteobacteria |
| Order | Acetobacterales | Deinococcales | Rhodobacterales | Acetobacterales |
| Family | Acetobacteraceae | Deinococcaceae | Rhodobacteraceae | Acetobacteraceae |
